# Maximum in the Middle: Nonlinear Response of Microbial Plankton to Ultraviolet Radiation and Phosphorus

**DOI:** 10.1371/journal.pone.0060223

**Published:** 2013-04-04

**Authors:** Juan Manuel Medina-Sánchez, José Antonio Delgado-Molina, Gunnar Bratbak, Francisco José Bullejos, Manuel Villar-Argaiz, Presentación Carrillo

**Affiliations:** 1 Departamento de Ecología, Facultad de Ciencias, Universidad de Granada, Granada, Spain; 2 Department of Biology, University of Bergen, Bergen, Norway; 3 Instituto del Agua, Universidad de Granada, Granada, Spain; University of Delaware, United States of America

## Abstract

The responses of heterotrophic microbial food webs (HMFW) to the joint action of abiotic stressors related to global change have been studied in an oligotrophic high-mountain lake. A 2×5 factorial design field experiment performed with large mesocosms for >2 months was used to quantify the dynamics of the entire HMFW (bacteria, heterotrophic nanoflagellates, ciliates, and viruses) after an experimental P-enrichment gradient which approximated or surpassed current atmospheric P pulses in the presence vs. absence of ultraviolet radiation. HMFW underwent a mid-term (<20 days) acute development following a noticeable unimodal response to P enrichment, which peaked at intermediate P-enrichment levels and, unexpectedly, was more accentuated under ultraviolet radiation. However, after depletion of dissolved inorganic P, the HMFW collapsed and was outcompeted by a low-diversity autotrophic compartment, which constrained the development of HMFW and caused a significant loss of functional biodiversity. The dynamics and relationships among variables, and the response patterns found, suggest the importance of biotic interactions (predation/parasitism and competition) in restricting HMFW development, in contrast to the role of abiotic factors as main drivers of autotrophic compartment. The response of HMFW may contribute to ecosystem resilience by favoring the maintenance of the peculiar paths of energy and nutrient-mobilization in these pristine ecosystems, which are vulnerable to threats by the joint action of abiotic stressors related to global change.

## Introduction

Inland water ecosystems are increasingly considered sentinels that provide signals of global change due to their high connectivity with terrestrial landscapes through transport and storage of water, energy and materials, besides harboring biodiversity threatened by human activity [1]. Within inland water ecosystems, high-mountain lakes, located in remote and high-altitude lands with small catchment areas, are exposed to extreme conditions (e.g. oligotrophy, high UVR fluxes) and are highly influenced by atmospheric processes. Because of their remoteness, high-mountain lakes provide excellent testimony on global change [2], [3] and are progressively gaining appeal as model ecosystems with simple biotic structures, but complex interactions, susceptible to rapid structural and functional changes in response to environmental perturbations [4]–[6]. Several major stressors related to global change, particularly affecting high-mountain lakes, have received attention. Ultraviolet radiation (UVR), a chronically acting stressor that directly harms organisms [7], has increased during stratospheric low-ozone events in northern latitudes [8] and, long after the Montreal Protocol, is still considered to be a global-change stressor because of the long time required for ozone recovery [9], [10]. Allochthonous loads of nutrients via atmospheric aerosol transport have increased in intensity and frequency of deposition due to land-use changes, and particularly over areas located near subtropical deserts (e.g. Mediterranean Region near Sahara desert), acting as sources of dust susceptible to atmospheric transport [11]–[13].

The few previous studies dealing with interactive effects of UVR and allochthonous nutrients on high-mountain lakes have reported a long-term massive development of UVR-tolerant non-flagellated algal species after experimental inputs of phosphorus in quantities close to current atmospheric depositions. This implies a drop in diversity and evenness due to a lower abundance of flagellated algae [14]. These findings are noteworthy, as most flagellated algae behave as mixotrophs which shape the entire planktonic structure and determine the energy pathway and nutrient mobilization to higher trophic levels not only in high-mountain lakes [2], [15], [16] but also in extensive oceanic clear-water areas [17]. Nevertheless, the interactive effects of abiotic stressors on HMFW have been scarcely reported, not only in high-mountain lakes but also worldwide, and those works are focused mainly on single components of HMFW, such as bacteria [18], [19] or ciliates [20]. Moreover, studies examining responses to the interaction of multiple stressors of the entire HMFW are even scarcer [21]–[23]. This scarcity is noteworthy given the key role of the HMFW, particularly in oligotrophic waters [24], [25], as a diverse network (composed of heterotrophic prokaryotes, protists, and viruses) processing and transferring auto- and allochthonous carbon and nutrients (from the dissolved pool) to higher trophic levels [26]. Because previous findings from high-mountain lakes showed that the joint effect of UVR and moderate P loads implies an increase in the availability of autochthonous organic carbon via algal excretion [27], [28], we hypothesize that greater nutrient availability linked to atmospheric loads [29] will stimulate the development of the HMFW. However, this stimulus will presumably be attenuated under ambient UVR, because of the widely reported direct negative effect of this factor on bacteria [7] and heterotrophic protists [30], [31]. The expected development of HMFW may imply the alteration of the pathways of energy and nutrient mobilization from bacteria to higher trophic webs by favoring the classical microbial loop against the bacteria-mixotroph link that is important in oligotrophic waters, such as high-elevation lakes [2], [15], [16] and extensive oceanic areas [17].

To test our hypothesis, we experimentally exposed a whole pelagic community (within large mesocosms) to a gradient of P enrichments that approximated or surpassed (i.e. acting as a stressor, [32]) current atmospheric nutrient pulses, in the presence and absence of ambient UVR, paying attention to the responses and relationships of virioplankton, bacterioplankton, and heterotrophic protists as components of the HMFW. This study is part of a long-term *in situ* experiment designed to examine how the interaction of global-change stressors (UVR × P loads) affects the entire pelagic food web in a model ecosystem. The responses of algal and zooplanktonic compartments are described elsewhere [12], [14], [27], while here we report the dynamics and response patterns of the HMFW, which reveal noteworthy ecological mechanisms and implications.

## Materials and Methods

### Study Site

The study was conducted in the high-mountain lake La Caldera, located above the tree line (3050 m above sea level) in Sierra Nevada National Park (southern Spain). The lake is described elsewhere [27]. Research permits for this study were provided by Sierra Nevada Parque Nacional (Spain).

### Experimental Set Up

We performed a long-term *in situ* experiment covering almost the entire ice-free period (1 August 2003 to 10 October 2003). The experimental setup is explained in detail elsewhere [27]. Briefly, it consisted of 10 mesocosms made of clear polyethylene tubes (0.7 m in diameter ×7 m in length, 2.7 m^3^). The mesocosm size and incubation time were fitted (i) to encompass body size and developmental cycles of all organisms in the entire pelagic trophic web subjected to the experimental conditions, (ii) to maximize the residual water volume per mesocosm during the last sampling (>85% initial volume), (iii) to allow organisms to migrate vertically (7 m in length). The mesocosm design resulted in a relatively high surface:volume ratio which might be of concern. However, wall colonization was detected (visual inspection) only at the end of the experiment and periphyton was thus not considered to have a significant impact on mesocosm plankton.

Mesocosm were filled with water pumped from 3 m deep (within the photic layer affected by >5% UVB). The experiment had a 2×5 factorial design with two light treatments, i.e. full sunlight (+UVR) and exclusion of ultraviolet radiation (−UVR), and with five nutrient treatments, i.e. adding inorganic P (as NaH_2_PO_4_) to a final concentration of 0 (control), 20, 30 40, 60 µg P L^−1^, and inorganic N (as NH_4_NO_3_) up to a N:P molar ratio of 30 to ensure that P remained a limiting nutrient. This nutrient gradient matched natural atmospheric P-inputs, as the 60 µg P L^−1^ treatment (P60) was about twice the measured maximum direct input in the lake [33] but lower than estimates corresponding to a single event of 81.37 µg P L^−1^ calculated from samples collected weekly from a nearby lake [4].

The +UVR treatment was applied using the polyethylene plastic that transmits 90% photosynthetic active radiation [PAR (400–700 nm)], and most UVR (60% UVB and 75% UVA). The −UVR treatment was applied using 2-m^2^ layers of Plexiglas UF3 (Atohaas Americas Inc, Philadelphia, PA, USA), a long-wave-pass plastic that transmits 90% PAR but blocks UVR (<390 nm), to envelope the entire rack of −UVR mesocosms in order to prevent any direct or indirect solar UV radiation. The top of each mesocosm was covered with a cap (made of polyethylene for +UVR and of Plexiglass UF3 for −UVR mesocosms) to avoid uncontrolled atmospheric nutrient inputs into mesocosms. After P enrichment, and before each sampling, the water of each mesocosm was mixed along its entire length and then integrated depth samples were taken. Mesocosms were sampled eight times during the 70-day incubation period (1 August 2003 to 10 October 2003) on days 1, 3, 11, 20, 32, 43, 56, and 70.

### Biomass of Microbial Plankton

Algal and bacterial phosphorus were determined from an aliquot of sample water from each mesocosm (100–300 mL), previously sieved through a 40-µm Nitex filter to remove zooplankton. The aliquot was subjected to serial filtration through precombusted (1 h at 550°C) 1-µm pore-size glass-fiber filter (Whatman GF/B, Kent, UK) and the filtrate through 0.2-µm pore-size polycarbonate filter (Nuclepore) at low pressure vacuum (<100 mmHg). Filters were introduced into acid-washed vials, digested with a mixture of potassium persulfate and boric acid at 120°C for 30 min, and determined as soluble reactive phosphorus in 10-cm quartz cuvettes by means of the acid molybdate technique [34]. Blanks and standards were performed for all procedures. The fraction 1–40 µm was identified as algae, and the fraction <1 µm as bacteria, because of the absence of size overlap between the different trophic levels, the lack of autotrophic picoplankton in La Caldera [15], [35], [36], and the negligible abundance of heterotrophic flagellates and ciliates previously observed in this lake [15] and corroborated in the present experiment, particularly after day 11. Variation in algal or bacterial phosphorus (namely ‘Δ-fraction P’) was calculated as the P content measured in each fraction and date divided by the P content in the corresponding fraction under the initial conditions.

Abundance and biomass of algae, bacteria, HNF, ciliates, autotrophic pico- and nanoplankton were quantified following the procedure described by Straškrabová, et al. [37]. Determination of ciliate abundance required disintegration of algal mucilaginous substances and to disaggregate lumps of algal cells. This was achieved by treating the samples with 3.7% HCl (final concentration) followed by an ultrasonic treatment for 10 min in an ultrasonic bath. The samples were then purified in an inverse flow filtration system that allowed the removal of particles <8 µm diameter. The purified sample was treated as described by Straškrabová, et al. [37]. Bacterial biomass was estimated from bacterial biovolume, measured from bacterial images obtained by transmission electron microscopy (TEM) as described by Medina-Sánchez, et al. [38].Virus abundance was quantified following the ultracentrifugation and TEM microscopy procedure described by Bratbak, et al. [39]. Viral biomass was estimated by using a conversion factor of 0.6 fg C virus−1 determined for viral particles with an average diameter of 60 nm [40].

### Algal Carbon Supply and Bacterial Production

Primary production (PP) and excretion of organic carbon (EOC) by algae were measured in triplicate for each experimental treatment and date as described by Carrillo, et al. [27]. After incubations, the organic ^14^C retained in algae (>1 µm, PP), bacteria (particulate organic carbon 0.2–1 µm, POC_b_, as autotrophic picoplankton <2 µm was absent), and dissolved fraction (dissolved organic carbon <0.2 µm, DOC) was separated by means of a serial filtration through 1- and 0.2-µm pore size, 25 mm diameter polycarbonate filters (Nuclepore Whatman). The algal excretion of organic carbon (EOC) corresponded to the organic ^14^C measured in the <1 µm fraction, i.e.
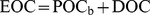



Bacterial production (BP) was measured following basically the procedure of Smith and Azam [41]. Briefly, sets of 12 (6 replicates +6 blanks) sterile microcentrifuge tubes per mesocosm and date, filled with 1.5 ml of the water sample and added with [methyl-^3^H] thymidine (TdR, specific activity: 2.6–3.2 TBq mmol^−1^, saturating final concentration: 11 nmol L^−1^) were incubated at in situ temperature in the dark for 1 h around noon. For the determination of the TdR incorporated only in bacterial DNA, incubations were stopped with NaOH (0.25 mol L^−1^, final concentration), causing basic hydrolysis of the ^3^H-labeled RNA. After extraction with 5% (final concentration) cold trichloroacetic acid for 20′ (leading to the precipitation of DNA and proteins), half of the tubes were subjected to an enzymatic digestion of the DNA with DNAse I (Boehringer Mannheim) solution (pH 7.5, at 37°C, for 2 h under gentle stirring). The TdR incorporated into DNA was calculated as the difference between the two treatments. BP was expressed in carbon terms using the conversion factors 1.07×10^18^ cells mol TdR^−1^ and 2×10^−14^ g C cell^−1^ calculated and used for this ecosystem [42]. Because bacterial respiration was not measured, we estimated the bacterial requirements for the photosynthetic carbon (preferentially used by bacteria in this ecosystem [35]) as CARB variable [28]. This was calculated as the quotient between BP and the bacterial use efficiency of the photosynthetic carbon. The latter term is analogous to bacterial growth efficiency because it measures the fraction of photosynthetic carbon (algal exudates) assimilated by bacteria:
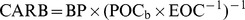



Because respired carbon was not included (not measured) in the POC_b_ term, the carbon supply (as 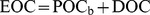) may be a lower estimate, while bacterial demands for this carbon (as CARB) an upper estimate of the respective actual values. Therefore, the comparison between EOC and CARB enabled us to assess whether the supply of organic carbon from algae met bacterial demands under the most restricted situation for each experimental treatment and period.

### Statistical Analysis

The effects of P enrichment on structural and functional response variables (i.e. abundance of bacteria, ciliates, and virus, HMFW biomass, algae:bacteria ratio, Δ-fraction P for algae and bacteria, BP) for each light condition (+UVR, −UVR [as ‘light’ control]) were assessed by linear or non-linear regression analysis of the response variable vs. P enrichment. When regressions were linear, the effect of ambient UVR was tested by analysis of covariance; whether the slopes of the regression lines were different for each light treatment (i.e. interaction with covariate, tested by homogeneity of slopes model, [43]), the UVR effect at particular P enrichment levels was graphically checked by examining 95% confidence intervals of the regression line (*see* [44]). When regressions were non-linear, the effect of ambient UVR was tested by paired *t*-test. The dependence of the UVR effect on P enrichment (interaction with covariate) was graphically checked by examining 95% confidence intervals of the regression line, which enabled us to test the UVR effect at particular P-enrichment levels [45]. The effect size of UVR or P was quantified as the quotient between the values for the treatment and the control, for each response variable.

Because the functional variables (e.g. BP, EOC, CARB) were measured in triplicate for each experimental mesocosm, Student’s *t*-test was used to check whether the organic-carbon supply from algae (EOC) met bacterial demands (CARB) for each experimental treatment; this approach was not used to test for a treatment effect (which would inflate the number of degrees of freedom available for this test; [46]), but to compare supply and demands for carbon per each experimental unit. Normality (by Shapiro-Wilks’ W-test) and homoscedasticity (by Levene’s test) were checked to ensure that the assumptions of parametric tests were met and, when not, the data were log transformed. Statistical analyses were performed using Statistica 7.1 (StatSoft Inc.) and OriginPro 8 SR4 (OriginLab) software.

## Results

### Dynamics of Microbial Plankton

**Figure 1:**
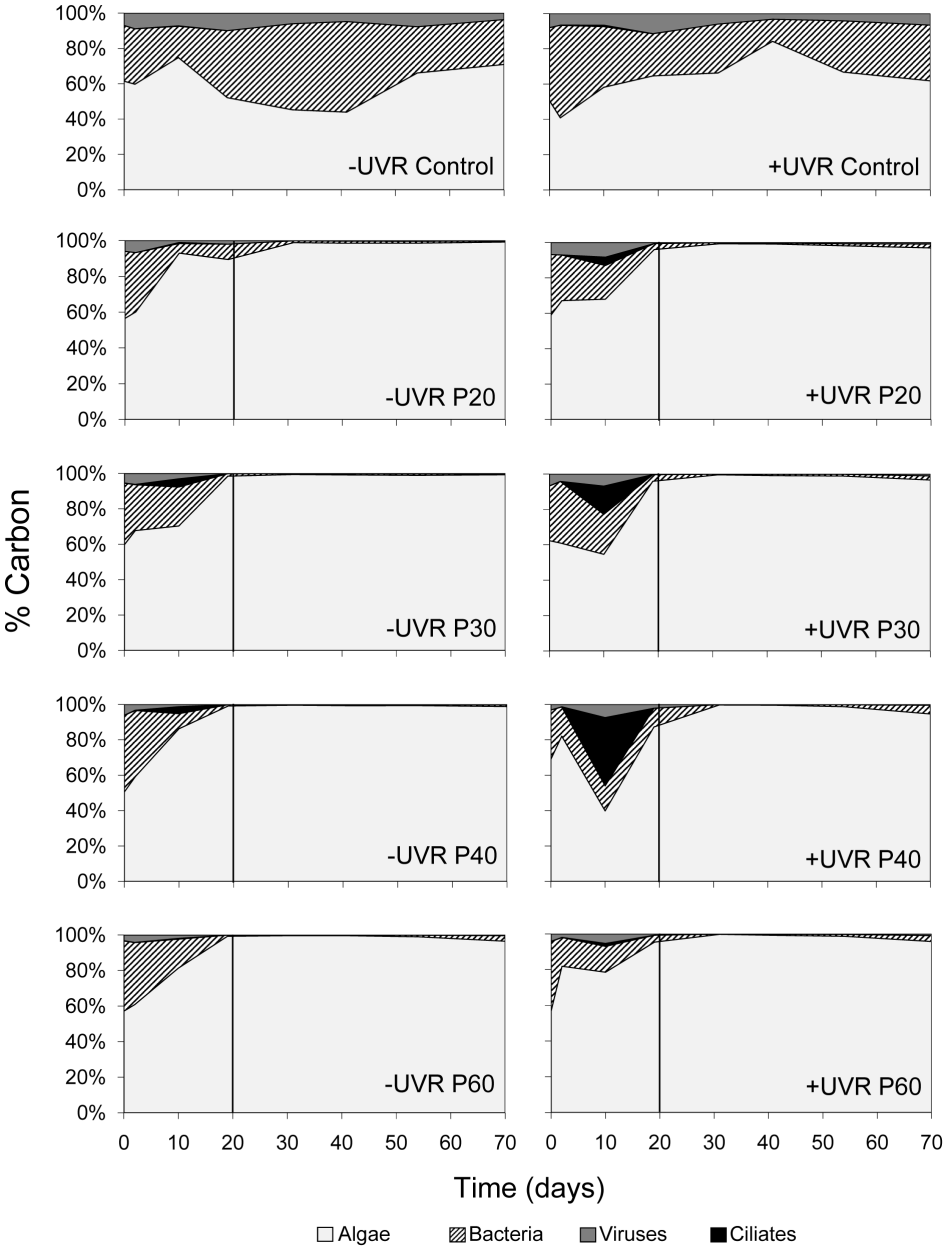
Biomass variation of each fraction of the planktonic community (<40 µm) over the experiment. Treatments with full sunlight radiation (+UVR; left panels) and without UV radiation (−UVR; right panels), as well as treatments without (Control) and with nutrient addition (20, 30, 40 and 60 µg P L^−1^) are displayed. The values are expressed as percentage of carbon. The vertical line divides experimental periods with or without availability of dissolved inorganic P.

Table 1 shows physical, chemical, and biological variables measured under initial experimental conditions. Based on the dynamics of inorganic P and N consumption [27] and on the temporal pattern of HMFW development over the experiment, a mid-term period (up to day 11) with inorganic P available (PA period) was distinguished from the rest of the period (from day 20 to the end of experiment) when the inorganic P was depleted (PD period). In mesocosms with no nutrient addition (controls), algae represented >50% of planktonic (<40 µm size) carbon biomass, whereas bacteria accounted for only about 30%, viruses represented a variable fraction of biomass (3–11%), ciliates were a minor fraction of nanoplankton (<1%), and HNF were absent throughout the experiment (Fig. 1). By contrast, in all nutrient-enriched treatments, algal carbon exceeded 90% of planktonic (<40 µm size) carbon biomass in the PD period (Fig. 1). Noticeably, the HMFW developed during the PA period, when ciliates reached a significant contribution to planktonic (<40 µm size) carbon biomass, mainly under +UVR (38%, Fig. 1). Thereafter (in the PD period), HMFW rapidly diminished and bacteria remained a minor fraction of planktonic (<40 µm) carbon biomass (<5%, Fig. 1).

**Table 1:** Physical, chemical, and biological variables measured under initial experimental conditions.

Variable	Value
T (°C)	12.3
Air UVB_300–318_ (W m^−2^)*	3.36
Air UVA_320–398_ (W m^−2^)*	46.0
K_d_ UVB (m^−1^)*	0.23
K_d_ UVA (m^−1^)*	0.15
TP (µg P L^−1^)	7.37
TDP (µg P L^−1^)	2.71
Algal-P (µg P L^−1^)	1.80
Bacterial-P (µg P L^−1^)	1.10
Algal abundance (cell mL^−1^×10^3^)	11.14
Algal biomass (µg C L^−1^)	15.90
Mixotrophic algae abundance (%)	>95
Bacterial abundance (cell mL^−1^×10^6^)	2.51
Bacterial biomass (µg C L^−1^)	10.69
Ciliate abundance (cell mL^−1^)	<1
HNF abundance (cell mL^−1^)	<1
Virus abundance (particles mL^−1^×10^6^)	3.89
Virus biomass (µg C L^−1^)	2.33
Algae:bacteria ratio (biomass)	1.49

Abbreviations: T, temperature (average water column); UVB_300–318_, ultraviolet B radiation measured in the 300–318 nm range (2 nm of interval); UVA_320–398_, ultraviolet A radiation measured in the 320–398 nm range (2 nm of interval); TP, total phosphorus; TDP, total dissolved phosphorus.

* UV radiation data were measured at noon using a LI-8000 spectroradiometer (LI-COR, Lincoln, NE, USA). Diffuse attenuation coefficients for downward irradiance (K_d_) were determined from the slope of the linear regression of the natural logarithm of downwelling irradiance vs. depth for each region of the solar-radiation spectrum.

### How UVR and Phosphorus Affected Biomass of Microbial Plankton

**Figure 2:**
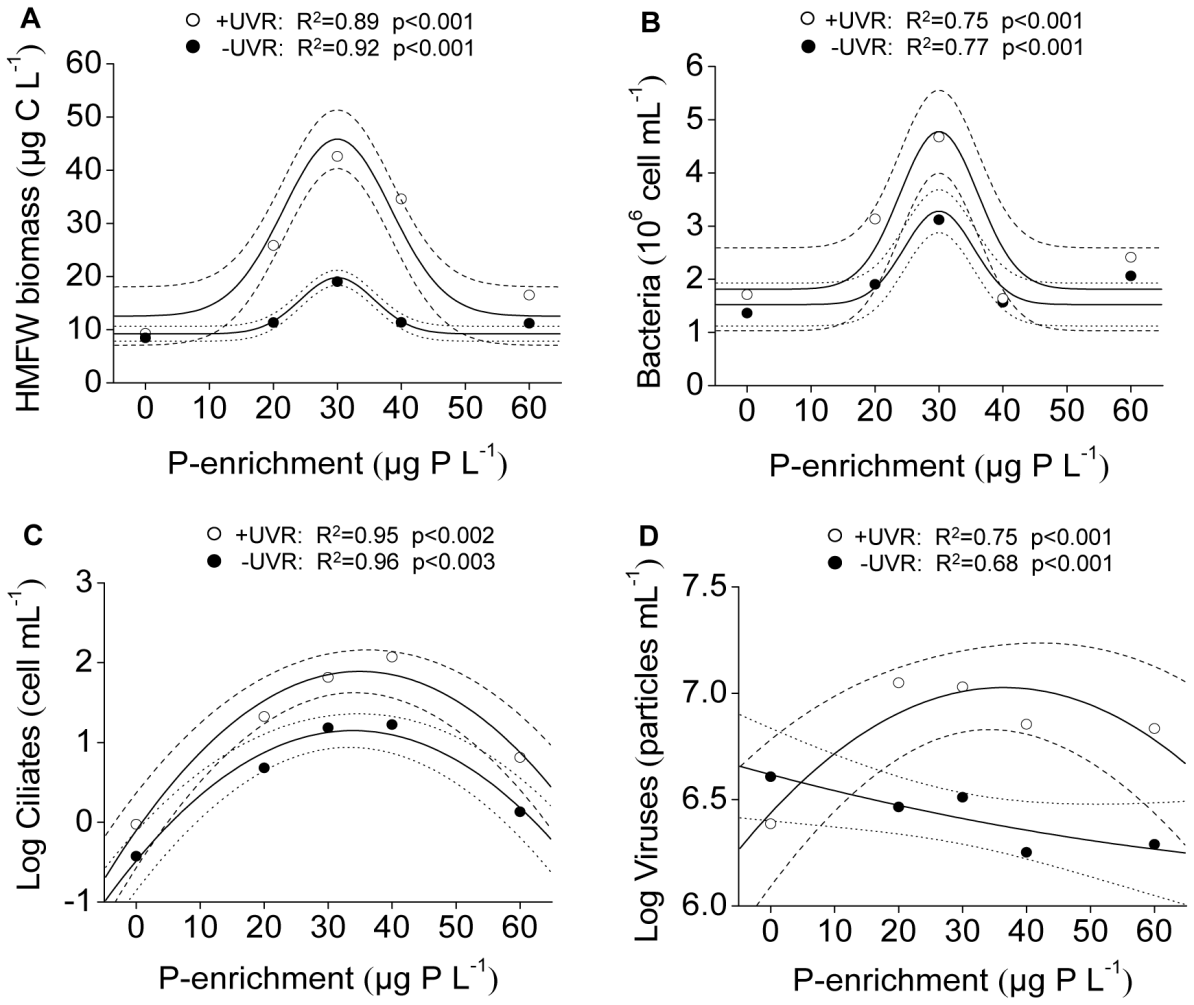
Non-linear regressions between HMFW components and P enrichment in the PA period. (A) Whole HMFW biomass; (B) bacterial abundance; (C) ciliate abundance; (D) virus abundance. Dashed and dotted lines indicate 95% interval confidence for each regression fitted by peak-Gaussian: 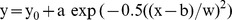 or quadratic: 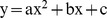 functions under +UVR and –UVR conditions. (PA period: phosphorus-availability period).

Besides the temporal pattern described above, the most notable result was that the whole HMFW biomass −as well as each of its components (bacteria, ciliates, and virus)− in the PA period showed a consistent unimodal response pattern along the P-enrichment gradient, peaking at intermediate P-enrichment levels (e.g. the effect size of P30 with +UVR was a 4.6-fold increase in biomass, Fig. 2A) and registering the lowest values in the control and P60 treatments. This general response was attenuated when UVR was excluded (e.g. the effect size of P30 was a 2.25-fold increase in biomass, Fig. 2A). The fact that 95% confidence bands of regressions for +UVR and –UVR treatments did not overlap at intermediate levels of P enrichment indicates that the effect of UVR was significant and maximum at these intermediate levels, and vanished toward the opposing ends of P-enrichment gradient. This implies a significant (p<0.05) interactive effect UVR×P enrichment. Bacterial abundance (BA) peaked in the +UVR/P30 treatment with a 2.7-fold increase (Fig. 2B). This increase was attenuated when UVR was excluded (1.8-fold). BA sharply decreased in the P40 treatments, yielding values similar (under +UVR) or slightly below (90%, under –UVR) those in the ambient control (Fig. 2B), coinciding with the greatest development of ciliate abundance in this P-enrichment level. The peaking of ciliates in the P40 treatment was sharper under UVR (125-fold) than with UVR excluded (17.8-fold) with respect to the ambient control (Fig. 2C). Viruses also responded acutely to P enrichment, reaching the maximum values in P20 (4.6-fold) and P30 (4.4-fold) under UVR, but the positive unimodal shape was not reflected in the absence of UVR (Fig. 2D). Overall, UVR exerted a positive effect on HMFW, bacteria, ciliates, and viruses, as indicated by the significant (paired *t*-test, p<0.05) increase for each compartment (i.e. >2.2-fold [HMFW], >1.5-fold [bacteria], >4.2-fold [ciliates] and >3.3-fold [viruses]) at intermediate P-enrichment levels (Fig. 2).

**Figure 3:**
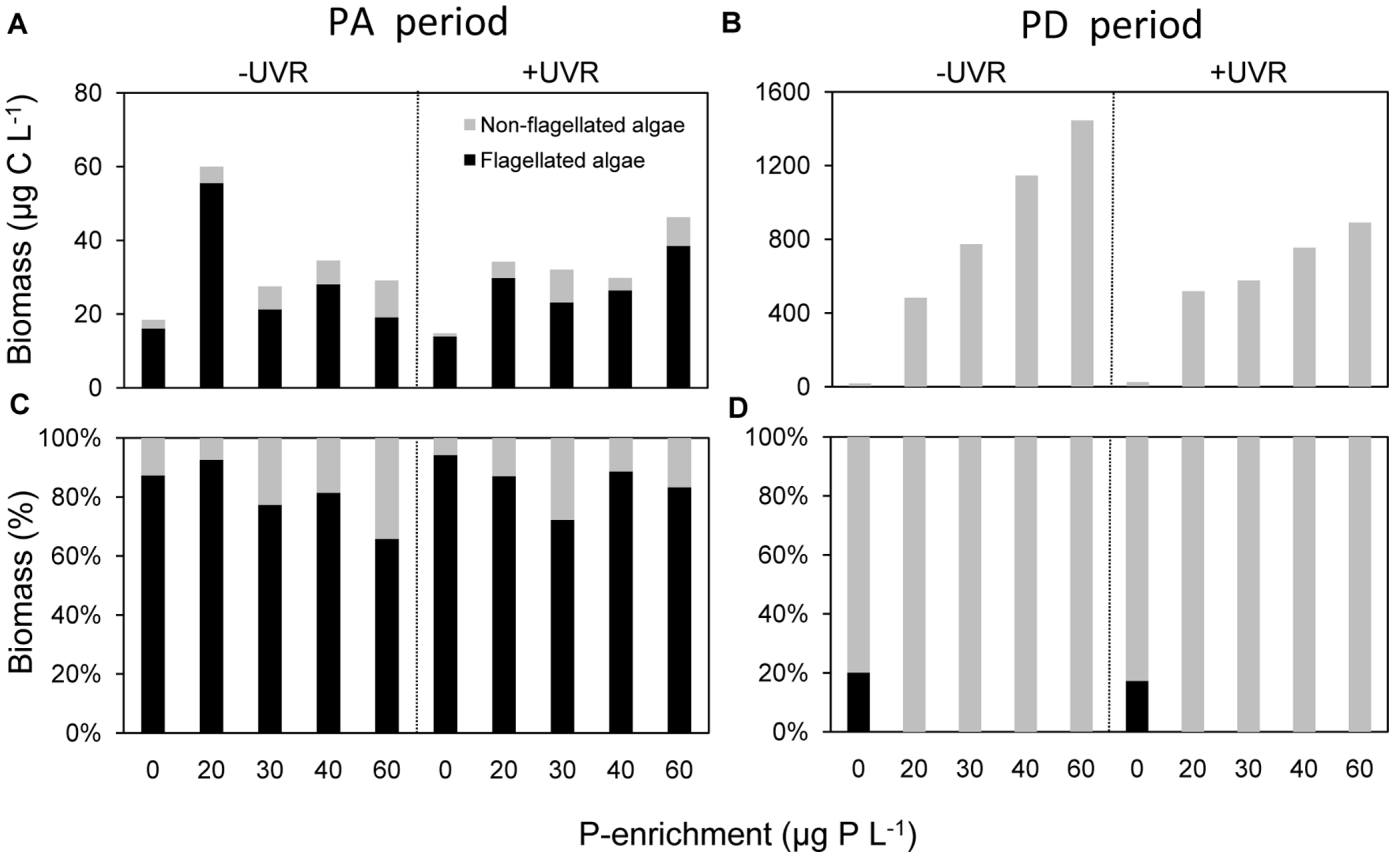
Responses of flagellated and non-flagellated algae to UVR and P enrichment over the experiment. (A) biomass absolute values for the PA period; (B) biomass absolute values for the PD period; (C) biomass percentage values for the PA period; (D) biomass percentage values for the PD period. Note the different scale on the Y-axis between the (A) and (B) panels.

During the PA period, the algal biomass was dominated by flagellates (mixotrophs) and responded to the P-enrichment gradient without showing the clear unimodal pattern of the HMFW components, particularly under +UVR conditions. By contrast, during the PD period, the algal biomass was dominated by non-flagellates, which strongly responded to the P-enrichment gradient, registering a clear linear increase, particularly sharp under −UVR (Fig. 3).

**Figure 4:**
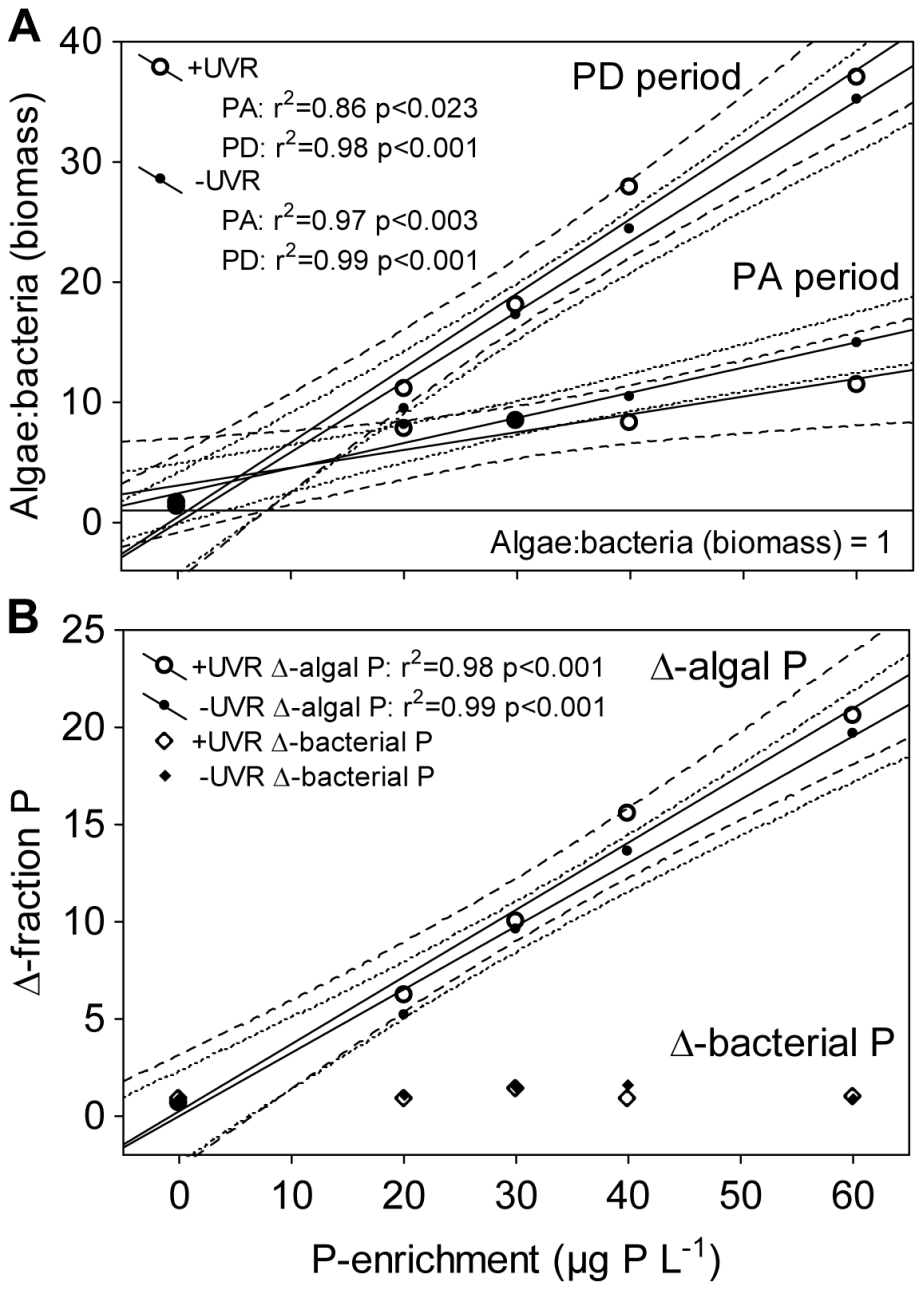
Linear regressions between structural variables and P enrichment. (A) algae:bacteria ratio (biomass) vs. P enrichment under +UVR and –UVR conditions in the PA and PD periods; (B) Δ-algal P or Δ-bacterial P vs. P enrichment under +UVR and –UVR conditions in the PD period. Dashed and dotted lines indicate 95% interval confidence for each regression. (PA period: phosphorus-availability period; PD period: phosphorus-depletion period).

The algae:bacteria ratio (in terms of biomass) was always >1 and showed a linear positive relationship with P enrichment (without UVR effect), reaching values up to 35 (+UVR/P60) in the PD period. The slope of algae:bacteria ratio vs. P enrichment did not vary between light treatments for each period (homogeneity of slopes model, p>0.5), but did increase significantly from the PA to the PD period (homogeneity of slopes model, p<0.0001; Fig. 4A). In addition, algae rather than bacteria immobilized most of the dissolved inorganic P. Thus, just after the collapse of HMFW (at the beginning of the PD period), when ciliate abundance was negligible and did not interfere with the algal-P measurement, the Δ-algal P linearly increased with P-enrichment (e.g. up to 15-fold the initial values in the P60 treatments), showing significantly higher values than Δ-bacterial P at any P-enriched treatment. Δ-bacterial P barely varied along the P-enrichment gradient, even yielding values beneath the initial bacterial P (Fig. 4B).

**Figure 5:**
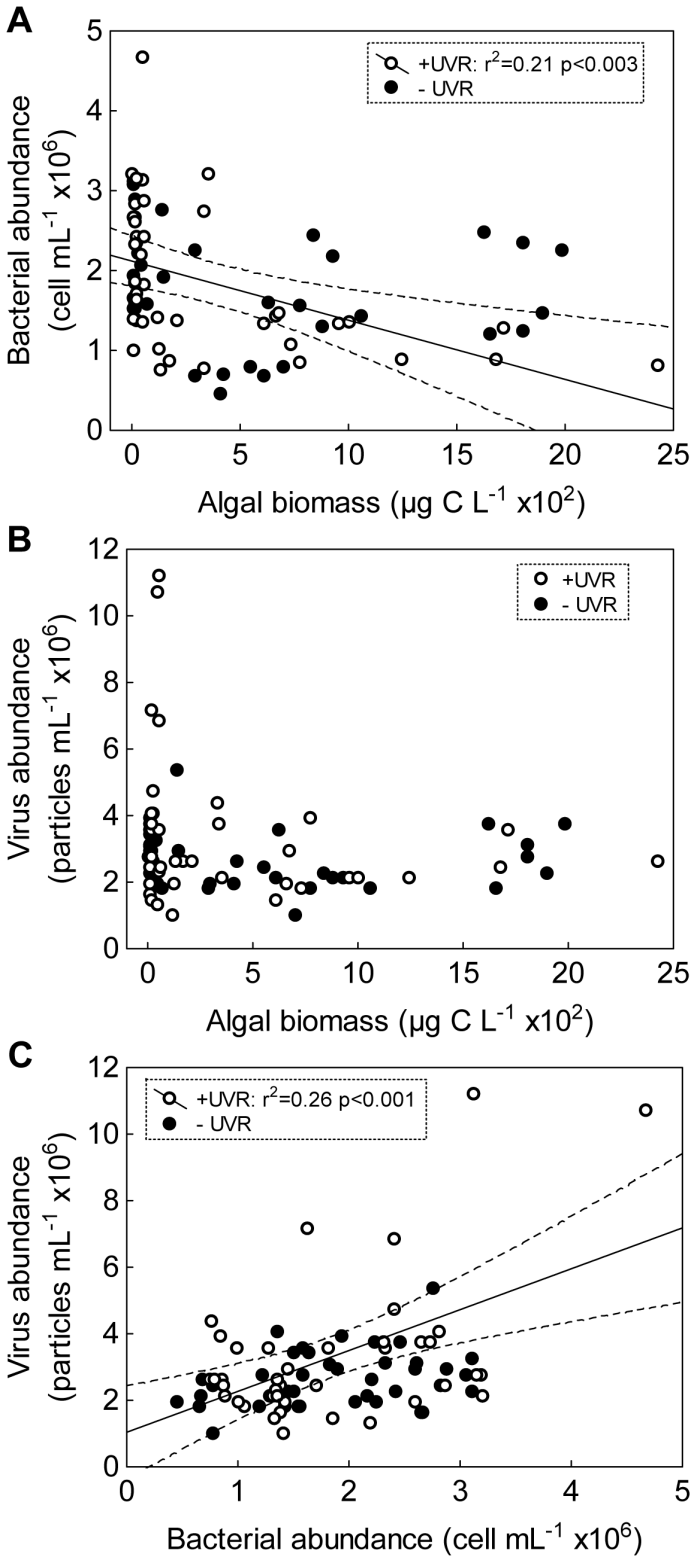
Relationships between compartments. (A) Bacterial abundance vs. algal biomass; (B) virus abundance vs. algal biomass; (C) virus abundance vs. bacterial abundance. Dashed lines indicate 95% interval confidence for each regression when significant.

Finally, over the experiment, bacterial abundance negatively correlated with algal biomass under +UVR (Fig. 5A); meanwhile, virus abundance did not correlate to algal biomass under any of the light conditions (Fig. 5B) but positively correlated to bacterial abundance only under +UVR (Fig. 5C). The virus-to-bacteria ratio (VBR) was generally low (from 0.6 to 4.6) but was significantly higher under +UVR than –UVR not only throughout the experiment (paired *t*-test, p<0.0003) but also in the PA period (paired *t*-test, p<0.015 considering the P-enriched treatments).

### How UVR and P affected Bacterial Production

**Figure 6:**
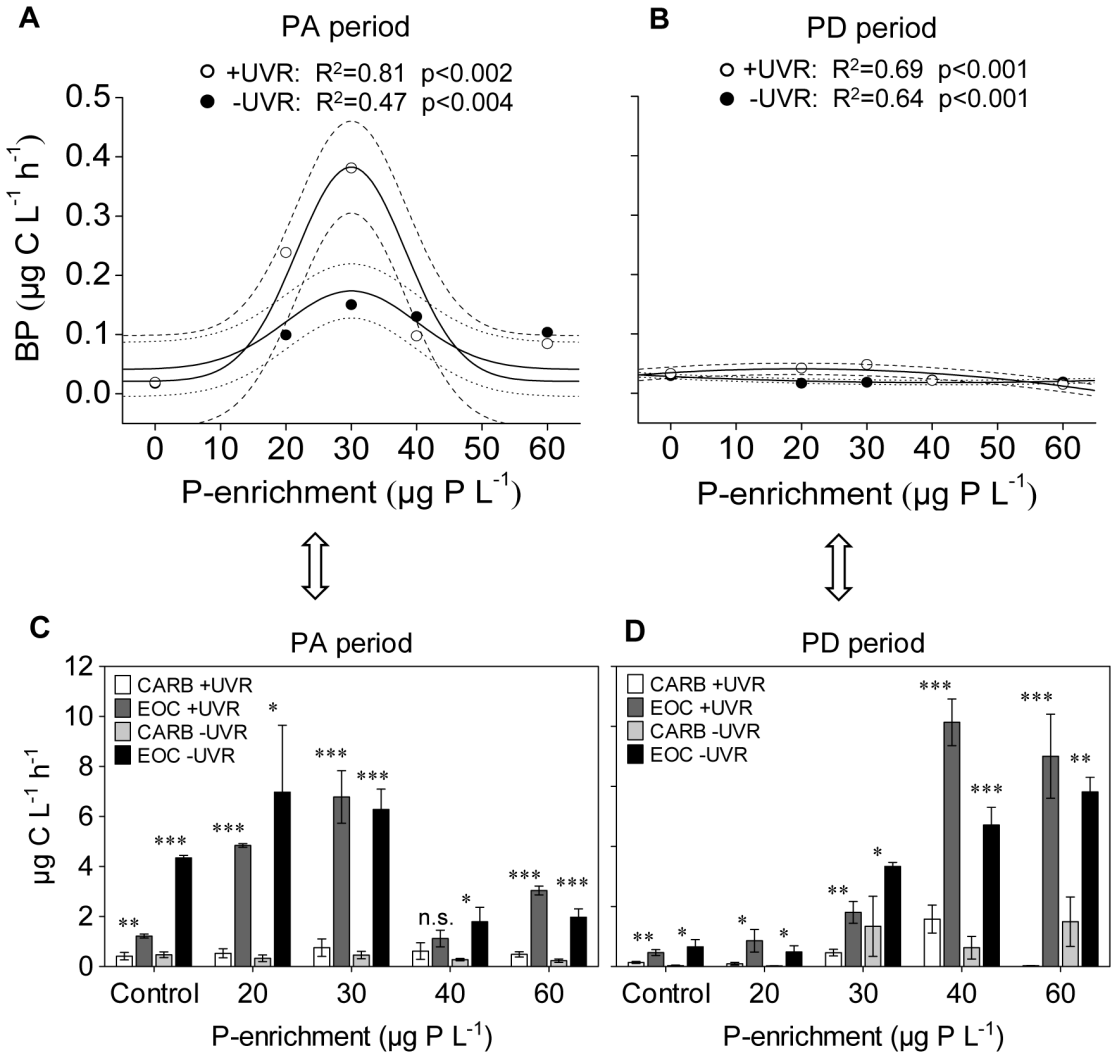
Non-linear regressions between BP and P enrichment, and bacterial requirements vs. supply of algal carbon. Dashed and dotted lines indicate 95% interval confidence for each regression, fitted as in Figure 2 in the PA period (A) and PD period (B). Photosynthetic carbon required by bacteria (CARB) and excretion of organic carbon from algae (EOC) measured in the PA (C) and PD (D) periods. Error bars are standard deviations. Significance of Student’s t-test between CARB and EOC for each experimental treatment: *p<0.05; **p<0.01; ***p<0.001. (PA period: phosphorus-availability period; PD period: phosphorus-depletion period).

Bacterial production showed a generalized response pattern to UVR and P enrichment similar to that of HMFW biomass in the PA period (Fig. 6A). The highest values were recorded at intermediate levels of P enrichment, and particularly under UVR (e.g. the effect size of P30 with +UVR was 19.8-fold), where the effect size of UVR was 2.4- and 2.5-fold in the P20 and P30 treatments, respectively (paired *t*-test, p<0.05). This pattern vanished during the PD period, when BP values decreased with the depletion of dissolved inorganic P (Fig. 6B).

**Figure 7:**
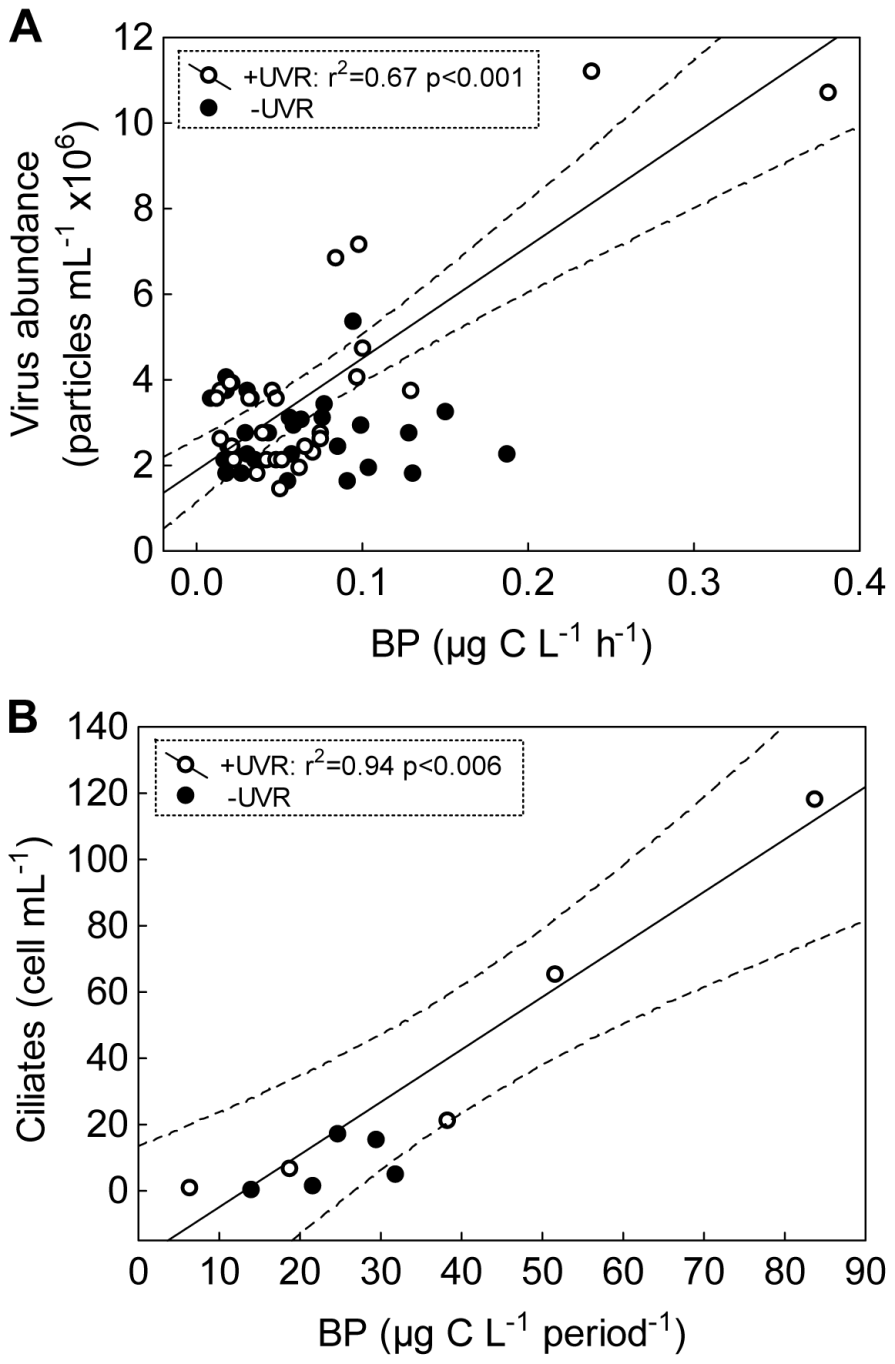
Linear regressions between structural and functional variables. (A) Virus abundance vs. BP; (B) ciliate abundance vs. total BP estimated for the PA period. Dashed lines indicate 95% interval confidence for each regression. (PA period: phosphorus-availability period).

The response pattern of BP to both factors was probably not constrained by the availability of organic carbon excreted by algae, since EOC met or exceeded the bacterial requirements for this carbon (CARB) to sustain BP under all enrichment and light conditions for each period (Fig. 6C,D). BP positively related to viruses, but only under +UVR (Fig. 7A). The developed population of ciliates in the PA period was positively related to total BP estimated for this period (which covers differences between bacterial and ciliate generation times), but only under +UVR (Fig. 7B).

## Discussion

This study, responding to the increasing demand for *in situ* experimentation to test non-additive effects of global-change stressors on ecosystems (e.g. [32]), reports how an entire HMFW responded to the joint impact of UVR and a P-load gradient which encompassed current and expected future scenarios of atmospheric dust deposition. The large spatial and temporal scales of the experimental set up allowed us to compile realistic information on entire trophic web dynamics [27] and infer their underlying ecological mechanisms through statistical analysis applied in mesocosm studies when experimental units could not be replicated [12], [44], [47]–[49]. Although the frequency at which the mesocosms were sampled precludes a detailed analysis for some compartments (e.g. viruses), it enables the quantification of the organisms’ first-order response to both factors at longer time scales.

### Communalities of Non-linear Responses to Resource Gradients within the Heterotrophic World

This study reveals an unexpected response pattern of a crucial compartment of aquatic ecosystems (HMFW) to the joint action of two major abiotic stressors linked to global change (high UVR fluxes and allochthonous P loads). The pattern consisted of an acute HMFW development over the mid-term following a unimodal function of the P-enrichment gradient, more pronounced under UVR, implying a significant UVR×P-enrichment interaction. The small increase in algal biomass under P-enriched conditions during the PA period, compared to their enormous increase in the PD period along the P-enrichment gradient, precludes a notable bias of the UVR×P-enrichment interaction on HMFW due to shading by the algae in the PA period.

The unimodal response of HMFW in PA period contrasts with the less clear pattern shown by (flagellated) algae, particularly under +UVR, and contrasts chiefly with the positive linear responses shown by non-flagellated algae to the same P-enrichment gradient during their acute development in the PD period (Fig. 3; [14], [27]). Our results constitute the first available report of unimodal responses of heterotrophic microbes to a nutrient gradient, comparable to those observed for macroscopic heterotrophic organisms such as fish, crustaceans, mollusks or insects, in which growth diminishes with high P content in their food [12], [50]. Boersma and Elser [50] proposed that these responses stem from increased metabolic costs derived from unbalanced nutrient content, even in a direction towards excess of a limiting nutrient. It is thus noteworthy that these unimodal responses were detected not only in (osmotrophic) bacteria, relying directly on dissolved resources, but also in their predators and even in non-metabolic parasites (viruses). Finally, it bears pointing out that this common non-linear response to nutrient gradient shown by the performance of heterotrophic organisms shares a common pattern with the widespread Intermediate Disturbance Hypothesis [51].

### Ecological Mechanisms Underlying the Response Pattern

Both HMFW and BP showed a similar unimodal response to P enrichment over the mid-term. This finding and the fact that BP was positively related to virus and ciliates (under UVR) provide evidence that mobilization of energy through bacterial growth is the functional basis for HMFW development. Notably, the increase of BP was modestly translated to bacterial abundance, whereas ciliates and viruses were acutely stimulated at the intermediate P-enrichment levels. A stimulus of BP which led to a surge in heterotrophic consumers (and parasites) but not in bacterial abundance is indicative of a top-down control on bacteria, according to predictions of food-chain models [52]. In the present experiment, this control was exerted mainly by ciliates and virus, because HNF were absent, probably due to a higher advantage of mixotrophic flagellates over HNF in oligotrophic ecosystems under stressing conditions [15], [53]. Our findings agree with those showing acute development of ciliates on actively growing bacteria [54], [55], supporting the view of protist bacterivory as a major top-down control responsible of bacterial loss, particularly in UVR-stressed ecosystems [23], [28], [56]. In addition, according to previous studies [57], the lack of positive correlation between viruses and algae, but positive between viruses and bacteria, suggests that algae were not the main viral hosts and viruses were probably bacteriophages, therefore also contributing to the top-down control of bacteria.

The strong stimulus of BP at intermediate levels of P enrichment under UVR could be favored because bacterial growth, the basis of HMFW development, had not only available phosphorus but also fresh carbon released from algae (EOC). In fact, carbon-supply rates (EOC) met or surpassed the bacterial requirements for this carbon (CARB) in all treatments, even though bacterial requirements for carbon in relation to supply (EOC) tend to increase in these autotrophic ecosystems [58]. Previous studies in this ecosystem showed that this carbon is readily taken up by bacteria, which are dependent on it [35]. In addition, the availability of both carbon (EOC) and phosphorus could confer bacteria additional photoprotection, e.g. by promoting the energetically costly repair mechanisms dependent on ATP (excision repair) which, along with the action of UVA-dependent photorepair mechanisms, may efficiently counteract the UVB-induced photodamage [59]. As a result, bacteria may become tolerant to the high UVR fluxes in these clear-water high-elevation lakes [42], accounting for the positive bacteria response to UVR found here.

The development shown by ciliates and viruses at mid-term involves the stimulating effect of UVR and P enrichment on both compartments. Whereas there is some consensus about a positive effect of nutrient enrichment on ciliates and viruses mediated through the stimulation of bacteria as prey and host, respectively [55], [57], controversy remains concerning the UVR effect on these organisms. Nevertheless, the positive response of ciliates to UVR found here agrees with findings in other high-mountain lakes [60], [61]. Similarly, the positive UVR effect on viruses found here may be explained through the activation of lytic cycles, prompted by the stimulus of bacterial growth with P enrichment under UVR. Nevertheless, since BP was measured by the thymidine-incorporation method, a share of the BP may include the synthesis of virus particles (i.e. viral production; e.g. [62]). This would reinforce a common pattern of response to UVR and P of viral production and the virus abundance measured. The low virus-to-bacteria ratio (VBR) found in the lake, with values within the lowest range reported for high-mountain lakes [63], polar inland waters [64], or Antarctic sea ice [65], but well below those reported for polar lakes where lytic cycles dominate [66], suggests that the virus response could stem from a bacterial population rich in lysogens. Because elevated VBR is indicative of high rates of viral infection of the bacterioplankton [57], it is noteworthy that VBR was significantly higher under +UVR than –UVR. This finding, as well as the positive relationship between virus abundance and BP found only under +UVR, suggests the ability of UVR to prompt lytic cycles on growing bacteria, in agreement with the general consensus [67], although few studies have tested this aspect in natural aquatic ecosystems (e.g. [68]). In summary, our findings suggest that organisms’ responses to global change stressors are propagated through interactions across multiple levels of organization [69].

### Microbial Heterotrophic vs. Autotrophic compartment: a Contrasted Regulation

The bolstering of the HMFW found at mid-term vanished over the long term (PD period) coinciding with an increase in the dominance of algae over bacteria, as reflected by the steeper regression slope of the algae:bacteria ratio vs. P enrichment in the PD period. This response is compatible with a competitive advantage of algae over bacteria (basis of HMFW) to immobilize P, related to their higher ability to store P when nutrient availability rises [36], [55], [70]. This competitive advantage is supported by (i) the significantly higher Δ-algal P values with respect to those of bacterial-P for each enrichment treatment; (ii) the higher slope of the linear fitting of Δ-algal P compared to that of Δ-bacterial P vs. P enrichment. Overall, our results suggest that the development of HMFW, enhanced at moderate P-enrichment levels under UVR, was constrained by direct biotic controls, mainly predation/parasitism over the mid-term, and competition with algae for P throughout the experimental period indicated by the negative relationship between bacteria and algae. By contrast, the autotrophic compartment would be regulated mainly by abiotic factors, as revealed by the response pattern of their structural and functional variables to the P-enrichment gradient [14], [27]. Therefore, the biotic controls of HMFW may be particularly accentuated in clear-water ecosystems, such as many high-elevation lakes, which may explain their weakly developed HMFW compartment (e.g. [2], [38], [71]) in comparison with that expected from the general oligotrophic pattern [24], [25].

### Implications for the Ecosystem

The mid-term development of HMFW triggered by moderate P enrichment may be speculatively interpreted as a self-organization mechanism favoring ecosystem resilience. Under moderate P load (i.e. the intermediate P levels), the diversion of part of P towards a transitory development of HMFW may be a mechanism favoring the persistence of a planktonic community dominated by mixotrophic flagellates as the main mobilizers of energy (via primary production) and nutrients (via bacterivory) in many clear-water ecosystems, such as high-elevation lakes or large oceanic areas [15], [16], [17]. This self-organization interpretation is compatible with previous experimental findings showing a coexistence of ciliates and mixotrophic flagellates after moderate P pulses [55].

However, under scenarios of high external P loads (e.g. >30 µg P L^−1^) expected by a increase in frequency and intensity of atmospheric dust deposition, the weak development of HMFW would be insufficient to avoid a strong growth of low-diversity algal communities [14], which even can impair the development of their zooplanktonic consumers [12], resulting in a loss of functional biodiversity.
